# Fast frequency relocking for synchronization enhanced resonant accelerometer

**DOI:** 10.1038/s41378-022-00428-5

**Published:** 2022-09-01

**Authors:** Liu Xu, Yonghong Qi, Zhuangde Jiang, Xueyong Wei

**Affiliations:** State Key Laboratory for Manufacturing Systems Engineering, Xi’an Jiaotong University, Xi’an, Shaanxi 710049 China

**Keywords:** Electrical and electronic engineering, Sensors

## Abstract

Synchronization, as a unique phenomenon, has been extensively studied in biology, chaotic systems, nonlinear dynamics, quantum information, and other fields. Benefiting from the characteristics of frequency amplification, noise suppression, and stability improvement, synchronization has been gradually applied in sensing, communication, time keeping, and other applications. In the sensing field, synchronization provides a new strategy to improve the performance of sensors. However, the performance improvement is only effective within the synchronization range, and the narrow synchronization range has become a great challenge for the wide application of synchronization-enhanced sensing mechanism. Here, we propose a frequency automatic tracking system (FATS) to widen the synchronization range and track the periodic acceleration signals by adjusting the frequency of the readout oscillator in real time. In addition, a high-precision frequency measurement system and fast response control system based on FPGA (Field Programmable Gate Array) are built, and the tracking performance of the FATS for static and dynamic external signals is analyzed to obtain the optimal control parameters. Experimental results show that the proposed automatic tracking system is capable of static acceleration measurement, the synchronization range can be expanded to 975 Hz, and the relocking time is shortened to 93.4 ms at best. By selecting the optimal PID parameters, we achieve a faster relocking time to meet the requirements of low-frequency vibration measurements, such as seismic detection and tidal monitoring.

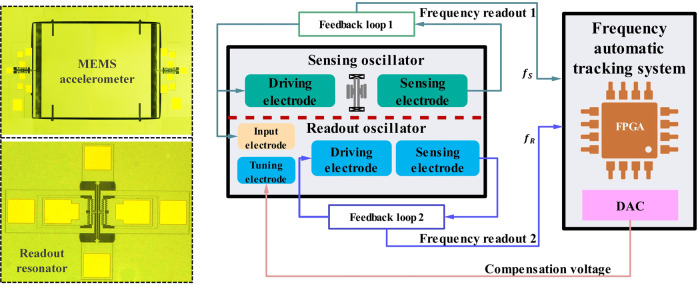

## Introduction

Synchronization is a phenomenon commonly observed in macroscopic coupled or driven oscillating systems^1^, e.g., in pendulum clocks^2–4^, metronomes^5,6^, and even celestial bodies^7,8^. Benefiting from the development of microelectromechanical systems^9,10^, micro/nanoscale resonators provide a platform of choice to test and demonstrate these concepts due to the experimental adaptability and possibility of accurate modeling. Micromechanical systems can achieve large nonlinearities at the origin of dynamics^11^. We note that synchronization has been investigated in single or coupled microelectromechanical systems with different frequency ratios^12–15^. Dario Antonio et al. experimentally demonstrated that a single autonomous oscillator synchronizes to an external perturbed force with a frequency of 1:1 and that the synchronization range increases with increasing oscillation amplitude under nonlinearities^16^. Matheny et al. investigated phase synchronization based on two anharmonic nanomechanical oscillators and a significant reduction in the phase noise of oscillators under the synchronization state^17^. Dong Pu et al. reported the synchronization of electrically coupled micromechanical resonators with a frequency ratio of 1:3, and the frequency stability of synchronized oscillators was improved nearly 10-fold^18^. In line with these latest studies, synchronization, as a new sensing mechanism, has been proven to improve the performance of micromechanical resonant sensors, such as sensitivity, accuracy, and resolution. Wang et al. first introduced the concept of a synchronization-enhanced sensing mechanism to an acceleration measurement field, and the zero-bias stability was improved two times compared to that without synchronization^19^. Subsequently, synchronization was achieved between a MEMS resonant accelerometer and an external DETF resonator by a unidirectional electrical coupling method, and the resolution was improved from 17.3 to 1.91 μg^20^. As a universal technique, the synchronized sensing mechanism can be utilized to optimize resonant sensors without compromising their original performance metrics.

However, the synchronization range directly determines the working bandwidth of the synchronized sensing mechanism, which critically limits the application and promotion of the synchronization phenomenon because the performance improvement is only proven effective in the locking region. Therefore, it is very important to find a way to expand the synchronization range. Here, we propose a frequency automatic tracking system (FATS) to achieve enduring locking between the MEMS resonant sensors and an external readout oscillator. When the target frequency exceeds the synchronization range, the locking state can be restored by adjusting the frequency of the external readout oscillator. This can track not only the static target frequency but also the dynamic target frequency (modulated frequency). In the first part of this paper, we describe the concept and the working regime of the synchronization sensing mechanism. To solve the narrow synchronization range, a frequency automatic tracking system is proposed to widen the synchronization range, and the response performance of the control system is assessed by transfer function analysis. Next, we achieved a synchronization sensing mechanism in experiments and built a high-precision frequency measurement and automatic tracking algorithm to verify the feasibility of the new strategy. Finally, we analyzed the tracking performance of the FATS for static and dynamic acceleration signals and selected the optimal parameters to improve the fast response ability of the control system. The proposed frequency automatic tracking system is also suitable for micromechanical resonant sensors, such as resonant accelerometers and pressure and force sensors.

## Working principle

The synchronization sensing mechanism^19–21^, as a new method to improve the performance of micromechanical resonators, has been used in the field of sensors, such as high-precision acceleration detection^19,20^ and weak capacitance measurement^21^. The synchronization sensing mechanism mainly consists of two elements, namely, a sensing resonator and a readout resonator. The sensing resonator can detect changes in the external physical environment and cause frequency shifts, such as acceleration, pressure, and capacitance. Its output signal is injected into the input electrode of the readout resonator as the perturbation signal. In the state of superharmonic synchronization, the frequency shifts of the sensing resonator can be amplified by the readout resonator to enhance the sensitivity. Generally, there are methods, such as mechanical coupling, electrostatic coupling, and unidirectional coupling, used to achieve mode interaction between the two resonators. In this paper, we utilize unidirectional electrical coupling to achieve superharmonic synchronization. Through personalized design, we make the frequency of the readout oscillator N times that of the sensing oscillator to meet the conditions of superharmonic synchronization. A real-time monitoring system is utilized to record the characteristic frequencies of the sensing resonator and readout resonator. According to our previous results, the stability of the readout resonator can be found to be enhanced in the synchronization state, so the sensor’s resolution can be improved. In addition, we propose a frequency automatic tracking system (FATS) to widen the synchronization range and track the periodic external signals by adjusting the frequency of the readout oscillator in real time.

**Fig. 1:**
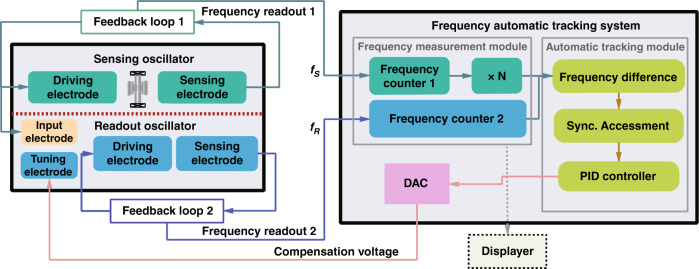
Schematic of the synchronized sensing mechanism and corresponding frequency automatic tracking system. The proposed tracking system consists mainly of a hardware module, i.e., a resonant accelerometer, an external readout resonator, a frequency monitoring system, and a software module, i.e., the synchronization state determining method, the electrical feedback, and the PID control

Figure 1 shows a schematic of the synchronization sensing mechanism and the corresponding frequency automatic tracking system. The frequency automatic tracking system is mainly composed of a frequency measurement module and automatic tracking module. The frequencies of the sensing oscillator (*f*_*S*_) and readout oscillator (*f*_*R*_) are measured by the frequency measurement module, and the control algorithm is executed by the automatic tracking module. The working principle of the automatic tracking module is as follows: First, the two frequencies (*f*_*S*_, *f*_*R*_) measured from the frequency measurement module are differentiated and compared with the preset threshold value. Once the frequency difference ($\delta f = f_R - N \cdot f_S$, where *N* is the synchronization order) exceeds the threshold, which means that the synchronization state is broken, the control system will trigger the frequency control module; otherwise, it will maintain the initial state. If triggered, the proportion integration differentiation (PID) controller will convert the frequency difference to the corresponding compensated voltage. The digital signal output by the automatic tracking module is converted into an analog voltage via a DAC (Digital to Analog Converter) and fed back to the tuning electrode of the readout resonator. The high-precision frequency measurement algorithms and the automatic control algorithms are written in the FPGA to realize the frequency measurement and automatic control of oscillators. The detailed control algorithm and transfer function analysis for FATS are shown in Supplement Section 1. Finally, the frequency-measured results are transmitted to the upper computer through the UART port for subsequent processing and display.

### Experiments and results

#### Test platform and devices

**Fig. 2:**
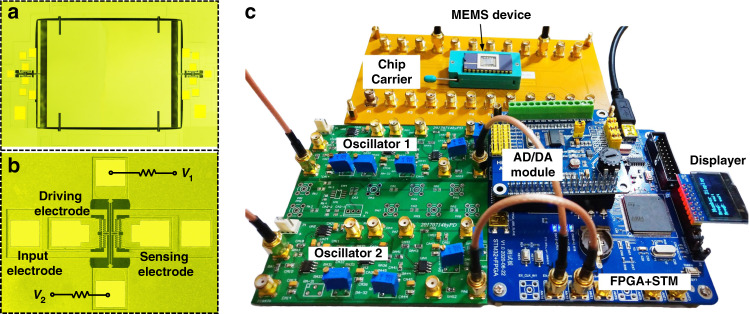
The experimental setup of the MEMS devices and frequency tracking system. **a**, **b** Microscopic graph of the MEMS resonant accelerometer and the external readout resonator. The micromechanical resonant accelerometer consists of a proof mass, two DETFs and microlevers. The proof mass is placed in the center, with the microlevers and the DETFs symmetrically arrayed around. **c** Photograph of the testing PCB (printed circuit board) and packaged MEMS resonant accelerometer. The synchronized resonant accelerometer is composed of a three-stage PCB circuit, including an oscillation circuit, FPGA, STM microcontroller, and AD/DA converter. The sensing resonator and readout resonator are self-oscillated by their oscillation circuit, and the stable oscillating frequency is input to FPGA for high-precision frequency measurement. The frequency automatic tracking algorithm is written into the STM microcontroller and controls the output voltage of the AD/DA module

Figure 2 shows the experimental setup of the synchronized resonant accelerometer and its corresponding frequency tracking system. In the synchronized resonant accelerometer we propose, the function of the sensing resonator is ‘actively’ detecting the external acceleration by frequency shifts, and the function of the readout resonator is ‘passively’ synchronizing with the sensing resonator and outputs its dynamic response. The MEMS resonant accelerometer consists of a proof mass, two DETFs (double-ended tuning forks) and microlevers. The proof mass is placed in the center, with the microlevers and the DETFs symmetrically arrayed around, which is shown in Fig. 2a. In the design process, we can adjust the resonant frequency of the sensing and readout resonator only by changing the length of the tuning fork under the condition that other structure parameters remain unchanged. In addition, finite element simulation software (COMSOL) is used to simulate the vibration mode frequency of sensing and readout resonators. The readout resonator in Fig. 2b is a DETF whose resonant frequency is designed to be three times that of the sensing resonator to meet the synchronization requirement. The micromechanical resonant accelerometer and the external readout resonator were fabricated using standard commercial (MEMSCAP) SOI-MEMS technology, including doping, metal lift off, patterning, and protection layer removal. After wafer fabrication and dicing, the MEMS devices were sealed in a ceramic DIP-24 package at a pressure of ~10 Pa by a metal cover and gold–tin solder. However, due to a manufacturing error, the resonant frequency of the fabricated sensing resonator and readout resonator is not perfect at 1:3, which will be overcome by the Joule heating effect^22,23^. The tunable frequency range of the readout resonator is ~15 kHz using electrothermal control, so a frequency difference within a range of 8.8% is acceptable. In the experiments, the sensing resonator of the resonant accelerometer operates in a linear state, while the readout resonator operates in a nonlinear state (Duffing nonlinearities) to enhance the synchronization range. The open-loop response of the sensing resonator and readout resonator are shown in Supplementary Section 2.

For the overall layout of the synchronized resonant accelerometer, the packaged device is mounted on a three-stage PCB, as shown in Fig. 2c. The first layer is used to lead the pins of the chip carrier with SMA connectors to facilitate the subsequent measurement. The second layer is the closed-loop feedback circuit module. The vibration signal of the sensing oscillator is picked up by the sensing electrode, and self-oscillation is realized through a closed-loop circuit. The second stage comprises front-end transimpedance amplifiers, a bandpass filter, a phase shifter, and a comparator. The phase shifter is used to ensure that the phase of the feedback loop meets Barkhausen’s phase criterion^24^. The bottom stage is the high-precision frequency measurement circuit based on FPGA. The multicycle synchronous frequency measurement method is applied to count the pulses on the rising edge of the square wave generated by the comparator. The frequency automatic tracking algorithm is written into the STM microcontroller and controls the output voltage of the AD/DA module. The algorithm can quickly calculate the required compensation voltage and output it to the tuning electrode of the readout resonator to complete a control cycle. The relationship between the compensation voltage and sensing oscillator’s frequency can be described by

1 $$\Delta V = \left| {V_1 - V_2} \right| = \frac{{\left( {R_0 + R_e} \right) \cdot \left( { - b + \sqrt {b^2 - 4a\left( {c - Nf_S} \right)} } \right)}}{{2a}}$$

where ∆*V* is the voltage difference of the double end of the DETF, *V*_1_ is the voltage of the first DC voltage source, *V*_2_ is the voltage of the second DC voltage source, *R*_0_ is the resistance of the resonator body, *R*_*e*_ is the external divider resistor, *a*, *b*, *c* is the thermal-frequency coefficient, and *N* is the synchronization order.

**Fig. 3:**
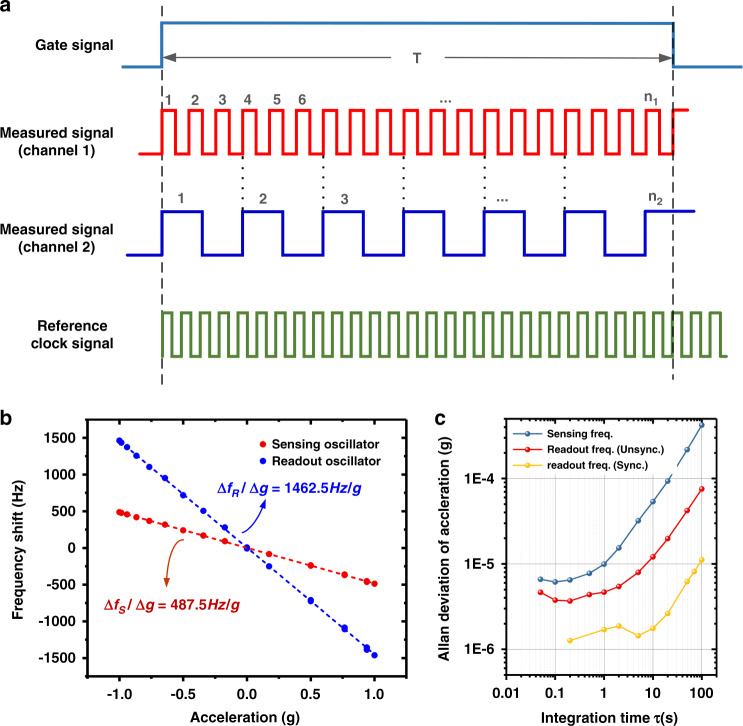
The experimental results of the multicycle synchronous method for high-precision frequency measurement based on FPGA. **a** Timing diagram of the multicycle synchronous frequency measurement method of the sensing oscillator (channel 2) and readout oscillator (channel 1). **b** The sensitivity enhancement results for sensing and readout oscillator. The frequency sensitivity of the sensing oscillator to tilt-induced gravitational loading is measured to be 487.5 Hz/g, while that of the readout oscillator is enhanced by three times to 1462.5 Hz/g under the synchronization state. **c** The frequency of the readout oscillator is 170,498 kHz, which is three times that of the sensing oscillator under the synchronization state. According to the calculation of the Allan deviation, the stability of the readout oscillator after synchronization was enhanced from 31.5 ppb to 12.4 ppb, which was an improvement of 2.54 times, while that of the sensing oscillator remained at 51.6 ppb. The calculated resolution of the original resonant accelerometer is 6 μg. However, with the help of synchronization enhancement, the resolution of the synchronized resonant accelerometer is increased fourfold to 1.45 μg

As mentioned before, the frequency measurement module uses the multicycle synchronous method to record the real-time frequencies. Figure 3a shows the time diagram of the multicycle synchronous method with reference clock signals. The frequency of the reference signals *f*_Ref_ is generated by the high-stability TCXO (50 MHz, XTALTQ). The gate signal is used to measure the oscillating frequency *f*_*x*_. The first rising edge of the measured signal triggers the gate signal to turn off once the *N*_*x* + 1_ rising edge arrives. Assuming that the count values of the reference clocks are *N*_0_, the frequency of the measured signal can be expressed by the following equation^25,26^:

2 $$f_x = \frac{{N_x}}{{N_0}} \cdot f_{\rm{Ref}}$$

The frequency measurement error is related to the gate time and the reference signal’s frequency. According to refs. ^25,26^, we can express the measurement error by:

3 $$\left| {\Delta f_x} \right| \le f_x/\left( {\tau \cdot f_{\rm{Ref}}} \right)$$

where *τ* is the gate time. Thus, using a more precise and stabilized crystal oscillator and extending the gate time will help to further improve the measured accuracy of FPGA-based multicycle frequency measurements. The oscillating frequencies of the sensing oscillator and readout oscillator are recorded by the frequency measurement module, and the frequency ratio of both oscillators is very close to 3:1. Measurement of the device sensitivity is carried out by placing the accelerometer on a tilt table, with the sensitive axis oriented at specified angles in 10° steps, as seen in Supplement Section 3. The frequency sensitivity of the sensing oscillator to tilt induced by gravitational loading is measured to be 487.5 Hz/g, while that of the readout oscillator is enhanced by three times to 1462.5 Hz/g under the synchronization state (Fig. 3b). Moreover, due to the unidirectional synchronization method, the temperature–frequency characteristics of the readout oscillator are exactly the same as those of the sensing oscillator (see Supplement Section 4). Figure 3c shows the experimental results of the Allan deviation of the two oscillators. The minimum Allan deviation of the readout oscillator under the free running state is 31.5 ppb at 0.2 s, which suppresses to 12.4 ppb at 5 s when synchronization occurs. Benefiting from the superharmonic synchronization effect, the sensitivity of the synchronization resonant accelerometer is enhanced by three times, and the minimum Allan deviation is reduced by 2.54 times. Thus, the theoretical resolution of our synchronized resonant accelerometer is enhanced to 1.45 μg compared with 6 μg of the original resonant accelerometer with synchronization.

#### Static tracking performance

As mentioned before, the performance improvement of our synchronization resonant accelerometer is only effective within the synchronization range. The narrow synchronization range severely limits the practical application. Fortunately, the proposed frequency automatic tracking system helps to dynamically extend the measurement range.

**Fig. 4:**
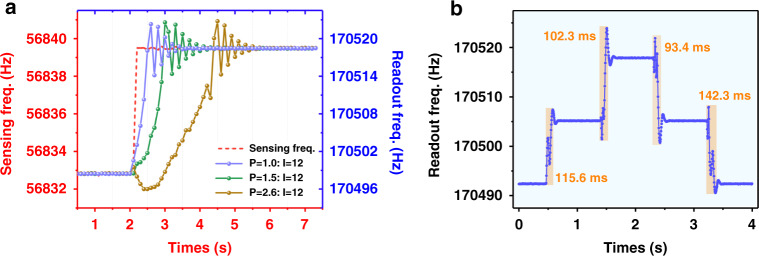
The experimental results of the frequency tracking system for the static accelerometer. **a** As the external acceleration changes by 13.7 mg (red dashed line), the readout oscillator’s frequency shifts three times stepwise with the sensing oscillator. Different PID parameters will affect the speed of frequency tracking. The experimental results corresponding to three series of PI parameters are represented by curves in different colors. **b** The tracking response of the readout oscillator for different external accelerations. By selecting the optimal PID control parameters, fast tracking of the readout oscillator is realized, and the shortest relocking time is 93.4 ms

Figure 4 shows the experimental results of the static tracking performance of the automatic tracking system. In Fig. 4a, the red dashed line represents the frequency output of the sensing oscillator with a pseudo acceleration of 13.7 mg, and the other lines represent the frequency output of the readout oscillator under the action of the control system. In the synchronization state, the frequency difference ($\delta f = f_R - 3 \cdot f_S$) is extremely small due to strict frequency matching. However, in the nonsynchronization state, there will be frequency detuning between the sensing oscillator and readout oscillator, so the frequency compensation module needs to be operated to lock the sensing oscillator’s frequency again. The value range of *δf* is 0~23 Hz. The PID system parameters directly affect the range and change rate of the frequency difference. The experimental results corresponding to three series of PI parameters are represented by curves in different colors. Taking the brown line as an example, during 0~2 s, the sensing oscillator and readout oscillator are under the synchronization state, and the frequency ratio of the two oscillators is 3:1 and remains constant. There is no need to execute voltage compensation during this time, and the DAC output voltage is constantly controlled at 2.0 V. Suddenly, the frequency of the sensing oscillator suddenly changes and exceeds the synchronization range at 2 s, and the readout oscillator’s frequency will no longer track the sensing oscillator. At the same time, voltage compensation is executed. Under the action of Joule heating, *f*_*R*_ will be dynamically adjusted to approximately three times of *f*_*S*_, and synchronization will occur again. From 2 to 4.2 s, the integral controller accumulates errors, causing the system to be in a deep saturation state. The system is in the saturation zone and hardly responds immediately to the real-time signal, which will cause the system to oscillate and overshot repeatedly until it stabilizes during 4.2–5.6 s. The overall relocking time of the system is 3.6 s. To solve the integral saturation phenomenon, our work replaces the traditional PI controller in the control loop with an integral separation PI controller. The working process of the integral separation PI controller is as follows: when the readout oscillator’s frequency (*f*_*R*_) deviates greatly from the reference value (3∙*f*_*S*_), only the P controller is activated to prevent the I controller from accumulating errors when the system’s output is saturated, which will cause the system to be in a deep saturation zone. When the readout oscillator’s frequency is close to the reference value, turn on the I controller to eliminate static errors and improve control accuracy. Figure 4a shows the experimental results of the tracking response with various PI parameters. As the P parameter increases, the total relocking time is also continuously shortened. Figure 4b shows the tracking response of the readout oscillator for different static accelerations. The relocking time is mainly affected by two aspects. One is the thermal response time of the resonator, which is determined by the material and structure of the readout resonator. The other is the PID control time. The speed of frequency tracking depends on the selection of PID parameters. A better PID parameter or control algorithm will shorten the tracking time and reduce overshoot. The shortest time is 93.4 ms in this work.

#### Dynamic tracking performance

**Fig. 5:**
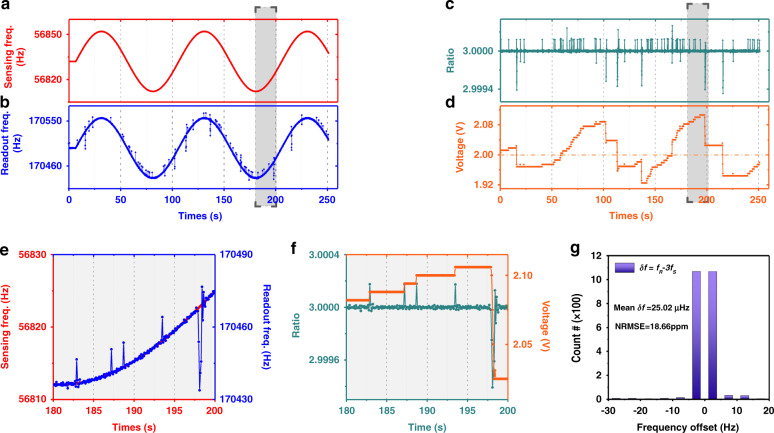
The experimental results of the frequency tracking system for dynamic low-frequency acceleration. **a** The output response of the sensing oscillator for modulated low-frequency acceleration (Ω = 0.01 Hz, ∆*a* = 82 mg). **b** The tracking response of the readout oscillator under the control of FATS. The amplitude of the readout oscillator is three times that of the sensing oscillator, which means that the sensitivity of the synchronized resonant accelerometer has been enhanced threefold. The readout oscillator can perfectly track and measure the dynamic environmental acceleration. **c** The frequency ratio between the sensing oscillator and readout oscillator. The ratio is constant at 3 in the synchronization state. Once the sensing oscillator’s frequency exceeds the synchronization range, the control system will execute immediately and quickly reach the synchronization state again. This process will lead to ‘glitches’. **d** The recorded compensation voltage of the DAC in the automatic control system. The overall trend lags behind the modulated acceleration signal. **e**, **f** The detailed expression of the sensing oscillator and readout oscillator corresponding to the gray rectangular plane. **g** The statistical distribution of the frequency difference between the sensing oscillator and readout oscillator of dynamic testing. The mean frequency difference *δf* is 25.02 μHz, and the NRMSE is 18.66 ppm, which means that the automatic control system can effectively track the dynamic modulation signal with great accuracy

The frequency automatic tracking system can not only quickly track the statically changing acceleration signal but is also suitable for tracking the dynamically modulated weak acceleration signal^27^. The resonant accelerometer was placed on the shaking table to detect low-frequency dynamic acceleration signals, while the readout resonator was statically placed beside and synchronized with the sensing resonator. The red line in Fig. 5a shows the response of the MEMS accelerometer for a dynamic low-frequency acceleration, similar to the frequency modulation signal. The modulated frequency is 0.01 Hz, and the amplitude is 82 mg. Thus, the dynamic modulation of the acceleration signal can be expressed as:

4 $$a\left( t \right) = a_c + \Delta a{\rm{cos}}\Omega t$$

where *a*_*c*_ is the fundamental acceleration, ∆a is the modulated amplitude of acceleration, and Ω is the modulated frequency. Figure 5b shows the frequency response of the readout oscillator under the action of the modulated signal. It can be shown that the readout oscillator can completely track the dynamic modulation signal. However, the blue line in Fig. 5b has many “glitches”. The reason is that when *f*_*S*_ exceeds the synchronization range, the automatic tracking system will execute immediately and quickly reach the synchronization state again. The process of frequency control leads to the “glitches”. Figure 5c shows the ratio of the sensing oscillator and readout oscillator. The ratio is constant at 3 in the synchronization state, and the “glitches” in Fig. 5b correspond to the jumping point in Fig. 5c. The output voltage of the DAC in the automatic control system is recorded in Fig. 5d. The output of the DAC changes continuously with the modulation signal, and the overall trend lags behind the modulated acceleration signal. In addition, the output voltage of the DAC changes stepwise, which is determined by the width of the synchronization range. Figure 5e, f shows the detailed outputs of the dynamic modulation signal and readout oscillator within 180–200 s. To estimate the frequency control error, the normalized root-mean-square-error (NRMSE) is used as a measure of the differences between the predicted and observed values^28,29^

5 $${\rm{NRMSE}}(f_R,f_S) = \frac{{\sqrt {\frac{1}{N}\mathop {\sum }\nolimits_{i = 0}^n \left( {f_R^{\left( i \right)} - 3 \cdot f_S^{\left( i \right)}} \right)^2} }}{{f_{R,0}}}$$

where *f*_*R*,0_ is the original frequency of the readout oscillator without acceleration. *N* is the sample number. $f_R^{(i)}$ and $f_S^{(i)}$ are the measured frequencies of the readout oscillator and sensing oscillator, respectively. NRMSE represents the difference between values predicted (3∙*f*_*S*_) and values observed (*f*_*R*_). When in the synchronization state, NRMSE will be extremely small due to frequency matching ($\delta f = f_R - 3f_S \approx 0$). When the synchronization is lost, the readout oscillator’s frequency jumps out of synchronization range and then synchronizes again under the action of FATS, which will introduce frequency control error ($\delta f = f_R - 3f_S\, \ne \, 0$). Therefore, NRMSE is used to measure the control accuracy of the frequency automatic tracking system. According to Eq. (5), we use a statistical distribution to analyze the dynamic modulated signal and the output of the readout oscillator, as shown in Fig. 5f. The mean frequency difference *δf* is 25.02 μHz, and the NRMSE is 18.66 ppm, which means that the automatic control system can effectively track the dynamic modulation signal with great accuracy. More tracking results for different modulation frequencies of the acceleration signal are shown in Supplement Section 5. A shorter relocking time will be beneficial for tracking higher frequencies of modulation signals for automatic tracking systems. Therefore, choosing optimal PID parameters and using faster processors will help track higher frequencies of modulation signals.

## Conclusion

In this work, we proposed a synchronization sensing mechanism to enhance the sensitivity and resolution of micromechanical resonant accelerometers. Benefiting from the superharmonic synchronization effect, the sensitivity of the synchronization resonant accelerometer is enhanced by three times, and the minimum Allan deviation is reduced by 2.54 times. Therefore, the resolution of our synchronized resonant accelerometer is enhanced to 1.45 μg as compared with 6 μg of the original resonant accelerometer with synchronization. However, the performance improvement of our synchronization resonant accelerometer is only effective within the synchronization range. Based on this, we propose a frequency automatic tracking system to widen the measurement range and track the periodic acceleration signals by adjusting the frequency of the readout oscillator in real time. In this way, the tracking system can expand the synchronization range to 975 Hz, and the relocking time is shortened to 93.4 ms at best. The proposed synchronization resonant accelerometer is suitable for low-frequency acceleration signal detection, such as structural mechanics, seismic detection and tidal monitoring.

## Supplementary information

Supplemental Material
