# Supplementary material for: Fast frequency relocking for synchronization enhanced resonant accelerometer
Source: Microsyst Nanoeng. 2022 Sep 1;8:93. doi: 10.1038/s41378-022-00428-5 (PMC9436963; doi:10.1038/s41378-022-00428-5)
Supplement: Supplementary file 1 — Supplemental Material [file 41378_2022_428_MOESM1_ESM.docx]

Supplement for ‘Fast frequency relocking for synchronization enhanced resonant accelerometer’

**1. Control algorithm and transfer function for FATS**

For the frequency automatic tracking system, one of the most important part is frequency control. When the frequency difference $\delta f$ between the two oscillators exceeds expectations, the manipulation needs to be activated to tune the frequency of one of the oscillators until the frequency difference meets the requirements again. The precisely frequency control is achieved by the programmable voltage based on the Joule heating effect. The positive and negative voltage are applied to the both ends of the double-ended tuning fork to generate the potential difference, which lead to the heat conduction and frequency change on the micro-resonator. In order to achieve fast and steady control, we need to analyze the transfer function of the control system. The voltage-temperature conversion module and temperature-frequency conversion module are shown in figure S1.


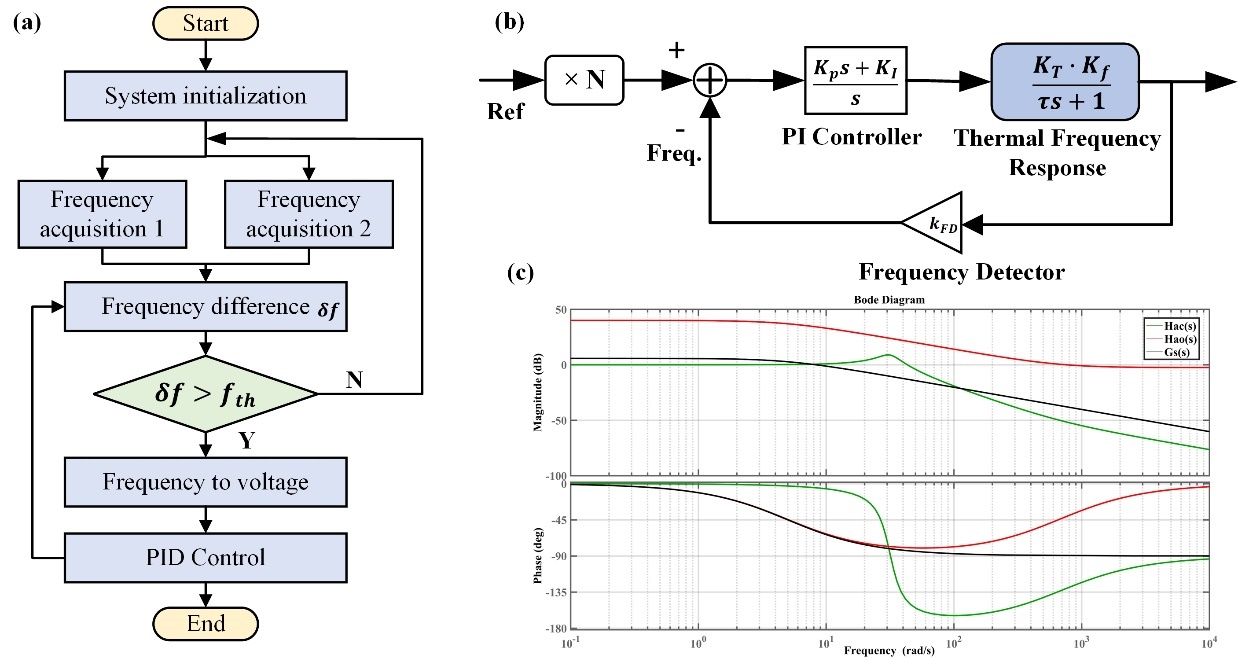


Figure S1. The algorithm analysis of frequency automatic tracking system. (a) The flow chart of the automatic tracking system. (b) The transfer function analysis of frequency feedback loop. The frequency feedback loop mainly consists of voltage-temperature conversion module and temperature-frequency conversion module. (c) The bode analysis of the thermal-frequency function $G_{s}(s)$, open loop gain function $H_{Ao}(s)$, and closed loop transfer function $H_{Ac}(s)$.

For the voltage-temperature conversion module, the thermal generated (dissipated) by the resonator body over a period of time will cause its temperature to rise (decrease), and the thermal balance equation can be expressed as

$\Delta Q=\Delta Q_{1}-\Delta Q_{2}$ (S1)

Where, $\Delta Q$ is the actual heat variation of the resonator within unit time, $\Delta Q_{1}$ is the heat generated of resonator within unit time, $\Delta Q_{2}$ is the heat dissipated of resonator within unit time.

The actual heat variation $\Delta Q$ is related to the thermal capacitance and the temperature changes of micro-silicon resonator,

$\Delta Q=C\frac{d\Delta\theta}{dt}=\frac{Cd(\theta-\theta_{o})}{dt}$ (S2)

Where, *C* is the thermal capacitance, $\theta$ is the temperature of resonator body, $\theta_{0}$ is the initial temperature, $\Delta\theta$ is the temperature change of resonator within unit time.

However, the heat dissipation on the surface of resonator $\Delta Q_{2}$ is related to the heat transfer coefficient, the area and the temperature difference between the resonator surface and the air (Assuming that the initial temperature of resonator surface is same as that of the air),

$\Delta Q_{2}=HA\left( \theta-\theta_{0} \right)=\frac{\theta-\theta_{0}}{R}$ (S3)

Where, *H* is the heat transfer coefficient, *A* is the area, $R$ is the thermal resistance.

Under the action of joule heating effect, the heat generation of resonator $\Delta Q_{1}$ have a nonlinear relationship with the voltage,

$\Delta Q_{1}=\frac{U^{2}}{r}$ (S4)

Where, *U* is the voltage, *r* is the equivalent resistance of the resonator. Linearization of above equation near the thermal balance point $\left( Q_{0},U_{0} \right)$ can be expressed as,

$\Delta Q_{1}=KU_{0}\Delta U=\frac{2U_{0}}{r}\Delta U$ (S5)

Substitute Eq. (S2), Eq. (S3) and Eq. (S5) into Eq. (S1), the thermal balance equation can be obtained by,

$\frac{Cd\left( \theta-\theta_{0} \right)}{dt}+\frac{\theta-\theta_{0}}{R}=\frac{2U_{0}}{r}\Delta U$ (S6)

Simplifying and transferring the above equation by the Laplace transform and setting the initial condition to zero, the transfer function $G_{1}\left( s \right)$ of the voltage-temperature conversion module can be expressed as,

$G_{1}\left( s \right)=\frac{\Delta\theta(s)}{\Delta U(s)}=\frac{K_{T}}{\tau s+1}$ (S7)

Where, $=RC$,$K_{T}=\frac{2RU_{o}}{r}$, *τ* is the time constant.

The temperature-frequency conversion module can convert the temperature changes of resonator into the frequency changes. The transfer function for this module represents the relationship between the temperature and output frequency. According to the frequency temperature response of the silicon microresonator, the transfer function $G_{2}\left( s \right)$ can be obtained as,

$G_{2}\left( s \right)=\frac{\Delta f}{\Delta\theta}=K_{f}$ (S8)

Therefore, the transfer function of thermal frequency response can be expressed as,

$H_{Ao}\left( s \right)=\frac{K_{f}K_{T}}{\tau s+1}$ (S9)

For the feedback control system, the sensing oscillator’s frequency $f_{s}$ is amplified by a frequency multiplier by N times, and compared with the readout oscillator’s frequency $f_{R}$ to obtain the frequency compensation value required by the PID controller. According to figure S1(b), the closed loop transfer function can be deduced by ($K_{T}K_{f}K_{p}\gg2$),

$H_{AC}\left( s \right)=\frac{K_{T}K_{f}(K_{p}s+K_{I})}{2\tau s^{2}+K_{T}K_{f}K_{p}s+K_{T}K_{f}K_{I}}$ (S10)

Where $K_{T}$, $K_{f}$ are the voltage-temperature conversion and temperature-frequency conversion coefficient, respectively. $K_{I}$, $K_{p}$ are the PID coefficient. From the Bode plot in figure S1(c), it can be seen that in the open loop state ($H_{Ao}(s)$), the bandwidth is narrow, which means that the response rate of the system cannot meet the practical requirement. If only use the open loop control, it will take more times for the system to reach a stable state, and the vibration is susceptible to external perturbation, which does not meet the response time requirements of the control system. The feedback loop can effectively solve the problems. The Bode plot of the loop gain $H_{Ao}(s)$ of the system shows that the PID controller effectively enhances the gain within the system’s bandwidth. Particularly, the integral term I makes the system’s gain raised at the low frequency, which is the key for controller to eliminate steady state errors.

The response of the system is divided into two states: transient and steady. The purpose of designing the control system is to make the stability of the system meet the requirements. The transient response quality should be high and the steady-state error of the system should be reduced. According to Eq. (10), the control system can be regarded as a second-order system, where undamped natural frequency $\omega_{n}$ and damping ratio $\xi$ are two important parameters. Assuming that the system frequency is $\omega_{n}^{2}=K_{T}K_{f}K_{I}/2\tau$ and system damping ratio is $2\xi=K_{T}K_{f}K_{p}/(2\tau\omega_{n})$, substitute it into the Eq. (S10) to obtain,

$H_{Ac}\left( s \right)=K_{T}K_{f}K_{p}\frac{1}{2\tau}\frac{s+\frac{\omega_{n}}{2\xi}}{s^{2}+2\xi\omega_{n}s+\omega_{n}^{2}}$ (S11)

So, the zero and pole point of system can be expressed by,

$z=-\frac{\omega_{n}}{2\xi}=-\frac{1}{b}$ (S12)

$p_{1,2}=-\xi\omega_{n}\pm\omega_{n}\cdot\sqrt{\xi^{2}-1}$ (S13)

Usually，the relative overshoot $\%OS$ and transition time $T_{s}$ are used to characterize the quality of transient response. The relative overshoot of the system is expressed as,

$\%OS=\exp\left( \frac{-\xi\pi}{\sqrt{1-\xi^{2}}} \right)\times100\%=\exp\left( -\pi\sqrt{\frac{K_{p}}{4K_{I}-K_{p}}} \right)\times100\%$ (S14)

The transition time can be expressed as (the steady-state deviation as 2%~3%),

$T_{s}\approx\frac{4}{\xi\omega_{n}}=\frac{16\tau}{K_{T}K_{f}K_{p}}$ (S15)

Figure S2 shows the change rate of frequency difference under different PID parameter states. It can be seen that with the increase of P parameter, the response speed of the control system continues to accelerate, and the changes rate of frequency difference increases from 11.44Hz/s to 51.07Hz/s, which is consistent with the trend of numerical simulation result. However, the P parameter cannot be increased indefinitely, otherwise the system will enter the deep saturation region, resulting in the accumulation of error in integral controller.


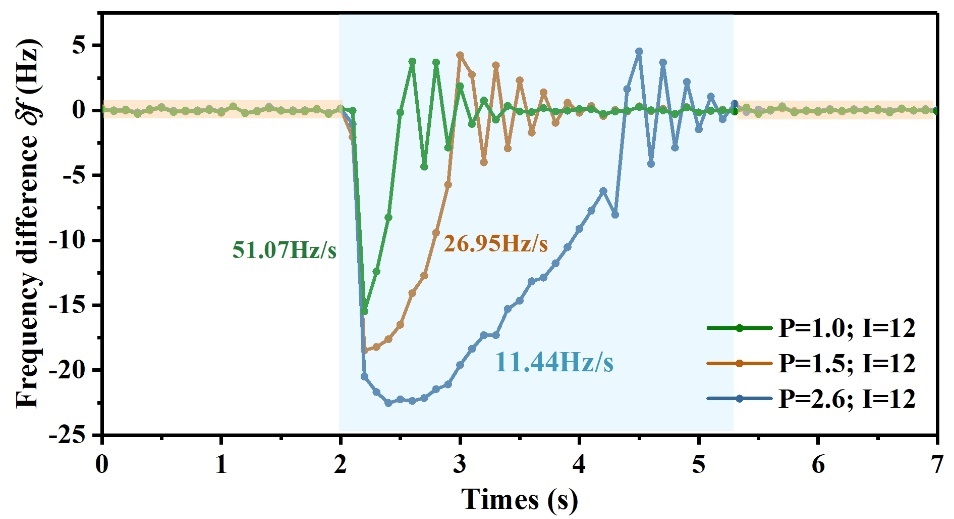


Figure S2. The experimental results of frequency difference $\delta f$ with different PID parameters.

**2. The schematic of synchronization enhanced resonant accelerometer**

The MEMS synchronized accelerometer was consisted of active sensing oscillator and passive readout oscillator. Driving pods were used to electrostatically actuate the resonators in flexural mode with $V_{ac}$ and $V_{dc}$. Sensing pods were applied to measure the vibration signal capacitively. In both oscillator systems, the closed-loop could provide the feedback signal to sustain the resonators vibration, which include trans-impedance amplifier, bans-pass filter, phase shift and comparator. The perturbation signal from sensing oscillator was injected into the input port of readout resonator for entraining the oscillators into synchronization. For frequency matching, the Joule heating effect was used to carefully tune the oscillator frequency of external resonator. Eventually, the frequency outputs of both oscillators were logged by the frequency measurement module and sent to frequency automatic tracking system to dynamically expand the working range. In experiments, the sensing resonator of resonant accelerometer operates in a linear state, while the readout resonator operates in a nonlinear state (Duffing nonlinearities) to enhance the synchronization range. The open-loop response of sensing resonator and readout resonator are shown (b) and (c).


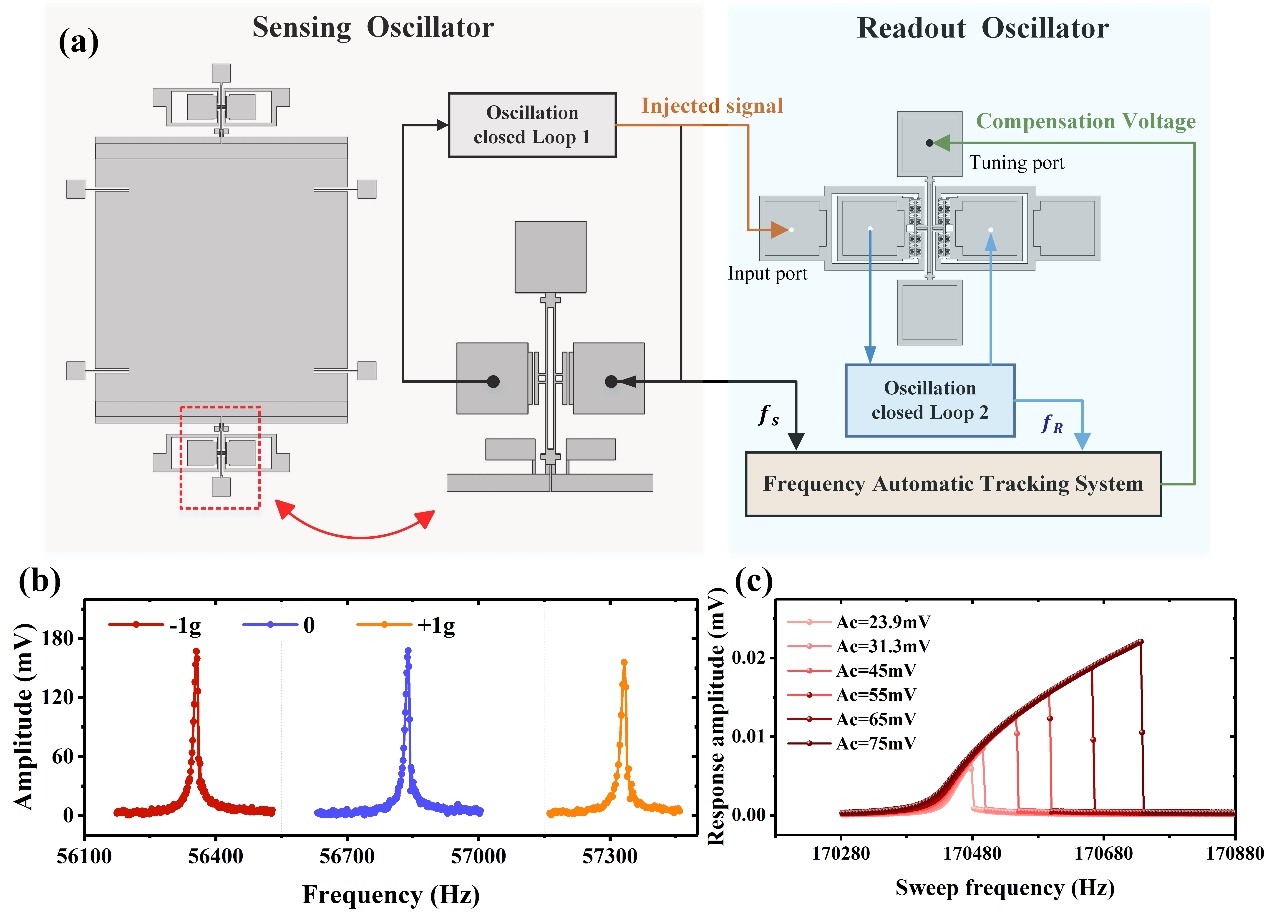

Figure S3. The measurement systems of MEMS synchronized accelerometer.

**3. Sensitivity**

The resonant accelerometer was installed on a rotary table to sense the acceleration change, while the readout resonator was statically arranged, shown in figure S4. The positional accuracy of the rotary table is 0.01°. We used the gravity field to measure the frequency shift of the sensing resonator of MEMS resonant accelerometer under the open-loop testing. After normalization, the installation error is eliminated and the measured sensitivity of resonant accelerometer is 487.5Hz/g.


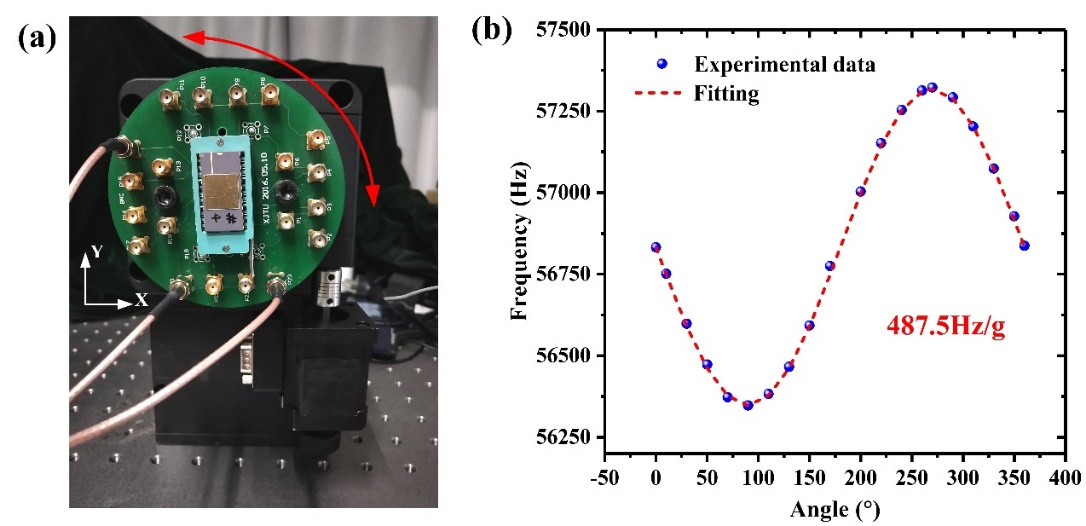


Figure S4. (a)The experimental setup of gravity field testing. (b)The experimental results of open-loop test with different angles.

**4. Temperature characteristic**

4.1 Setup and fundamental characteristics

We use the temperature control chamber to measure the temperature characteristics. The MEMS device was sealed in vacuum through a combo lid to provide a low damping ratio environment and ensure it can be worked normally. Then we placed it into the high precision temperature control chamber and connected the external test board (figure S5). Using the temperature controller, the temperature of the controlled region is ramped up from 0℃ to 100℃ with steps of 10℃. Figure S6 shows the experimental result of the continuous data collection of variable temperature experiment over full-temperature range. The temperature inside the chamber ramped down to 0℃ and up to 100℃. After that it was held a period of time to reach thermal equilibrium in at 100℃. The temperature was then ramped down to 0℃ to measure the frequency output continuously.


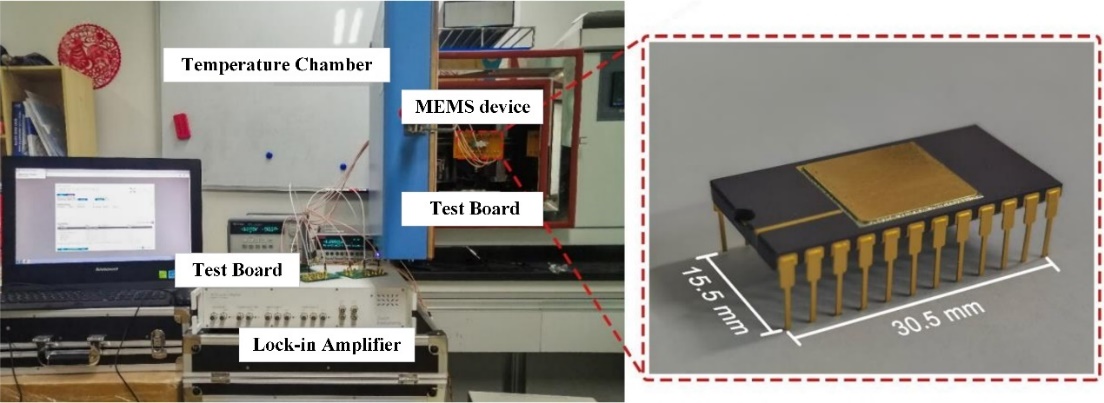


Figure S5. The experimental setup of the temperature characteristics.


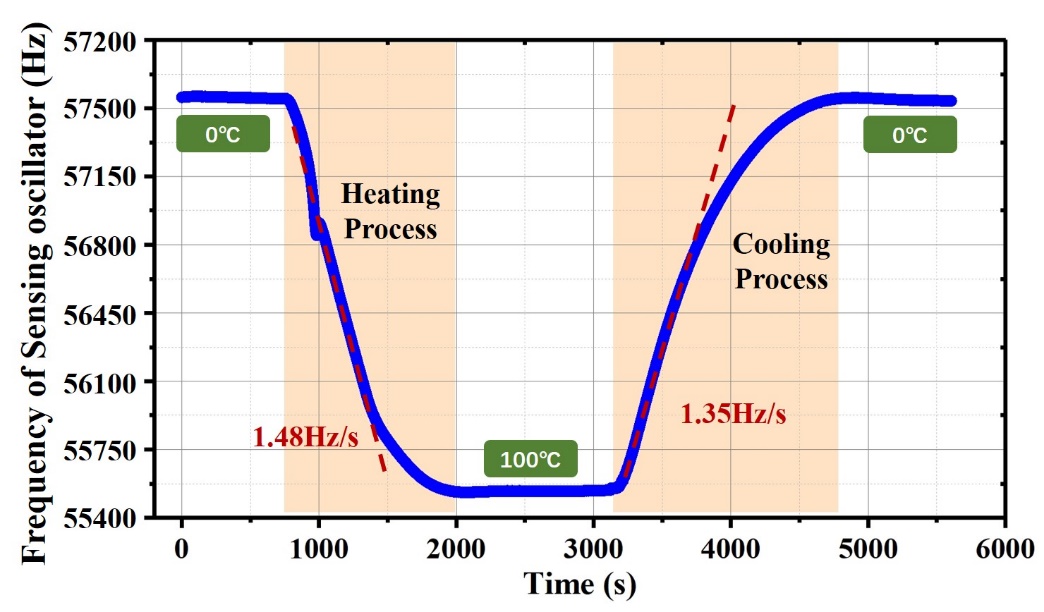


Figure S6. The continuous data collection of variable temperature experiment over full-temperature range.

The variation of the individual resonator frequencies and their frequency difference over temperature has been plotted in figure S7. The TCF is obtained by calculating the slope of the curves. The temperature-frequency characteristics of the readout oscillator are exactly the same as those of the sensing oscillator because the synchronization between the two oscillators is realized by means of unidirectional signal injection. The slopes of the two resonant frequencies are seen to be close but not identical to each other. Thus, the frequency difference has a residual temperature dependence. Due to this partial cancellation, the temperature induced drifts in a frequency difference output are not completely remove.


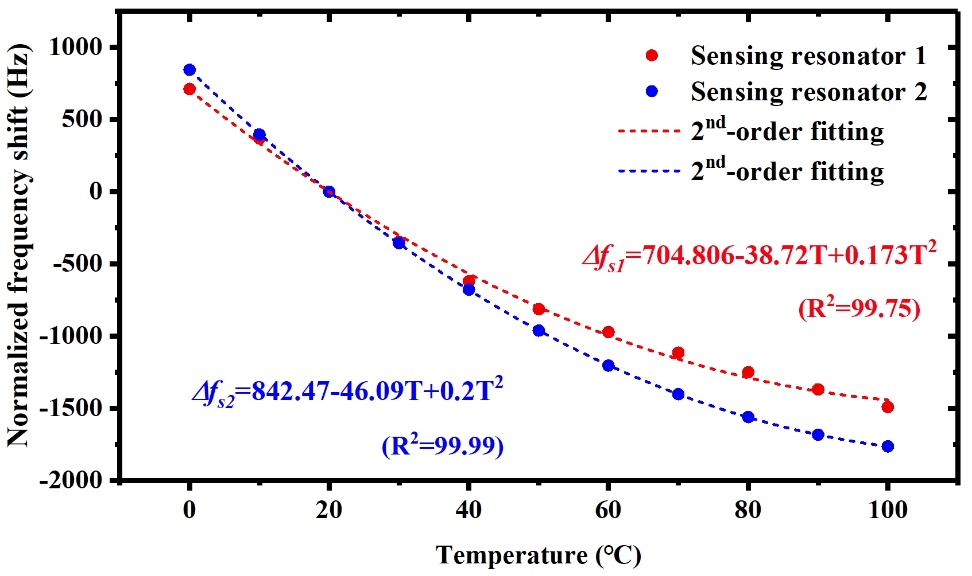


Figure S7. Temperature sensitivity characterization of the resonant accelerometer.

4.2 Temperature hysteresis effects

In order to explore the temperature hysteresis characteristics in MEMS resonators, experiment was performed to measure the frequency of sensing oscillator while the temperature was ramping up and down between 0℃and 100℃. The experiment of temperature hysteresis test was performed to measure oscillating frequency every 20℃ between the full temperature range from 0℃ to 100℃. The temperature in the chamber was increased in steps of 20℃, and sensing oscillator’s frequency was measured. The same process was repeated for decreasing temperature from 100℃ to 0℃. The experimental results are shown in figure S8. The result from these experiments indicates that the MEMS resonator have significant temperature hysteresis effects under ambient temperature variations, which may be related to residual stress and differential thermal expansion in the MEMS resonator.


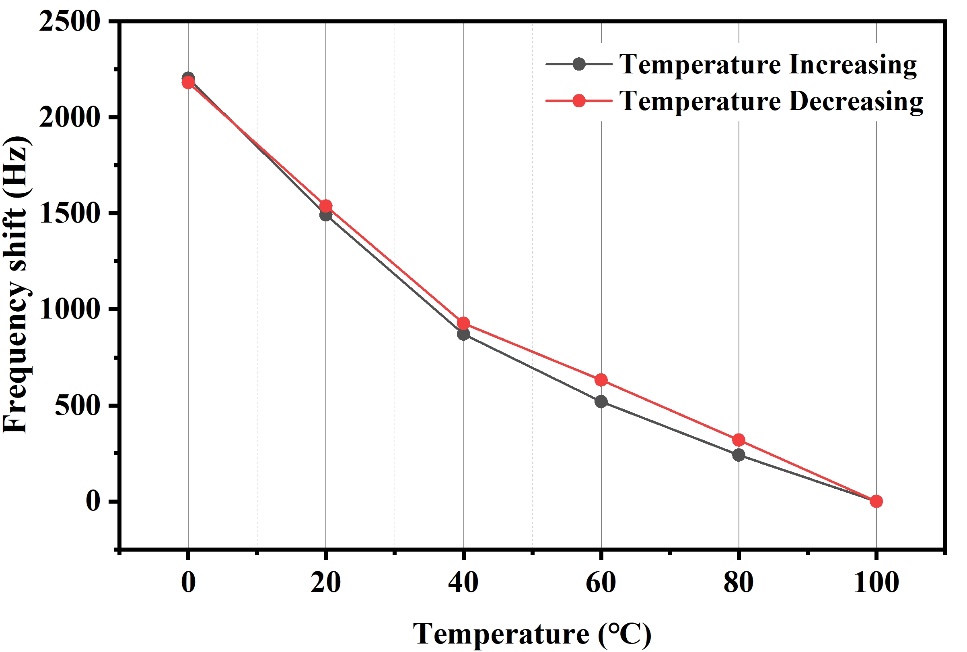


FigureS8. The test results of temperature hysteresis effect in MEMS resonators.

4.3 Temperature repeatability

For testing the temperature stability of the resonant accelerometer, repeatability experiments were carried out on the sensor in the full temperature range. The consistency of the temperature was evaluated by the frequency response after the sensor was turned off for 30 minutes. Selected different temperatures as measuring points (0℃, 20℃, 40℃, 60℃, 80℃, 100℃), and the experiment was performed for 6 times. The data was measured after the accelerometer was powered on for 10 minutes. The temperature in the chamber was increased in steps of 20℃, and at each step, the chamber was held for 10 minutes and allowed to reach thermal equilibrium. The experimental results are shown in figure S9 and Table 1. Thus, the temperature repeatability (3δ) of MEMS device is less than±0.107%.


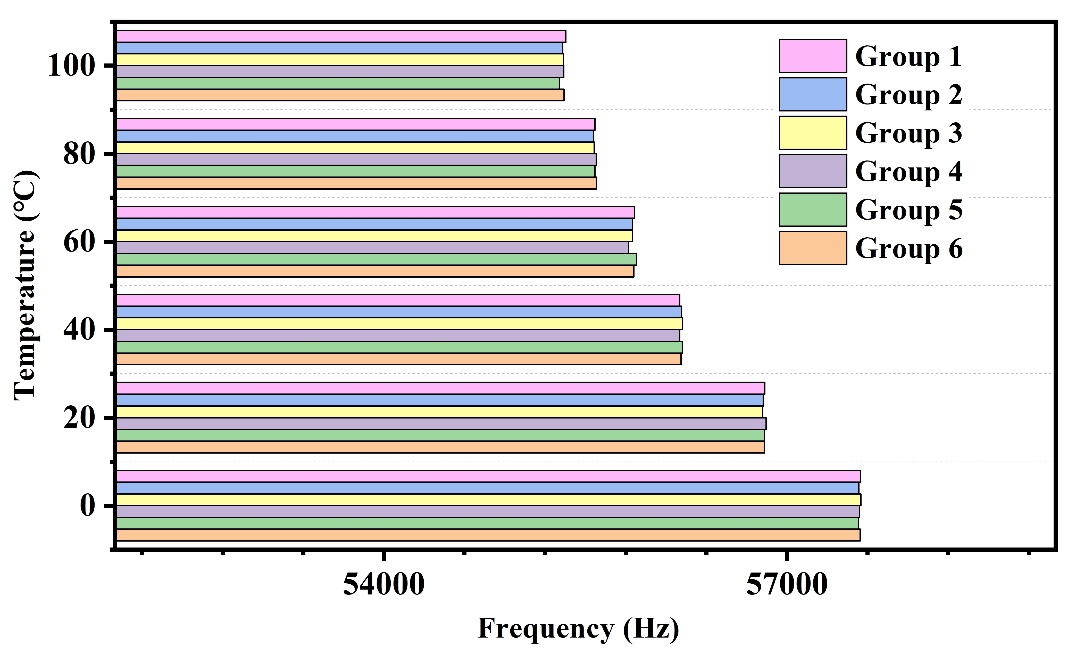


Figure S9. The experimental results of temperature repeatability test and temperature sensitivity characterization of the resonant accelerometer.

Table 1. The results of temperature repeatability test

|  | 0℃ | 20℃ | 40℃ | 60℃ | 80℃ | 100℃ |
| --- | --- | --- | --- | --- | --- | --- |
| Average (Hz) | 57542.22 | 56831.367 | 56211.89 | 55854.86 | 55571.99 | 55334.17 |
| Standard Deviation δ (Hz) | 7.800229 | 8.3650862 | 8.819893 | 19.90543 | 7.791795 | 16.03536 |
| Repeatability (3δ) | ±0.0406% | ±0.0442% | ±0.0471% | ±0.107% | ±0.042% | ±0.087% |

**5. Tracking for dynamic acceleration**

Figure S10 shows the tracking results for different modulation frequencies of acceleration signal. Figure S11(a), (b) and (c) represent the frequency of dynamic acceleration signal as 0.1Hz, 1Hz and 2Hz, respectively. As the modulation frequency increases, the dynamic modulation signal changes faster and faster, which requires the tracking time of automatic control system to become shorter and shorter. When the modulation frequency is 0.1Hz, readout oscillator’s frequency can completely track the frequency change of the modulation signal, and its NRMSE is 151ppm according to Eq. (6) in the manuscript. When the modulation frequency is increased to 2Hz, part of the frequency data will be out of tracking, and the NRMSE will increase to 300ppm. The shorter tracking time will benefit to track higher frequency of modulation signal for automatic tracking system. Therefore, choosing optimal PID parameters and using faster processors will help tracking higher frequency of modulation signals.


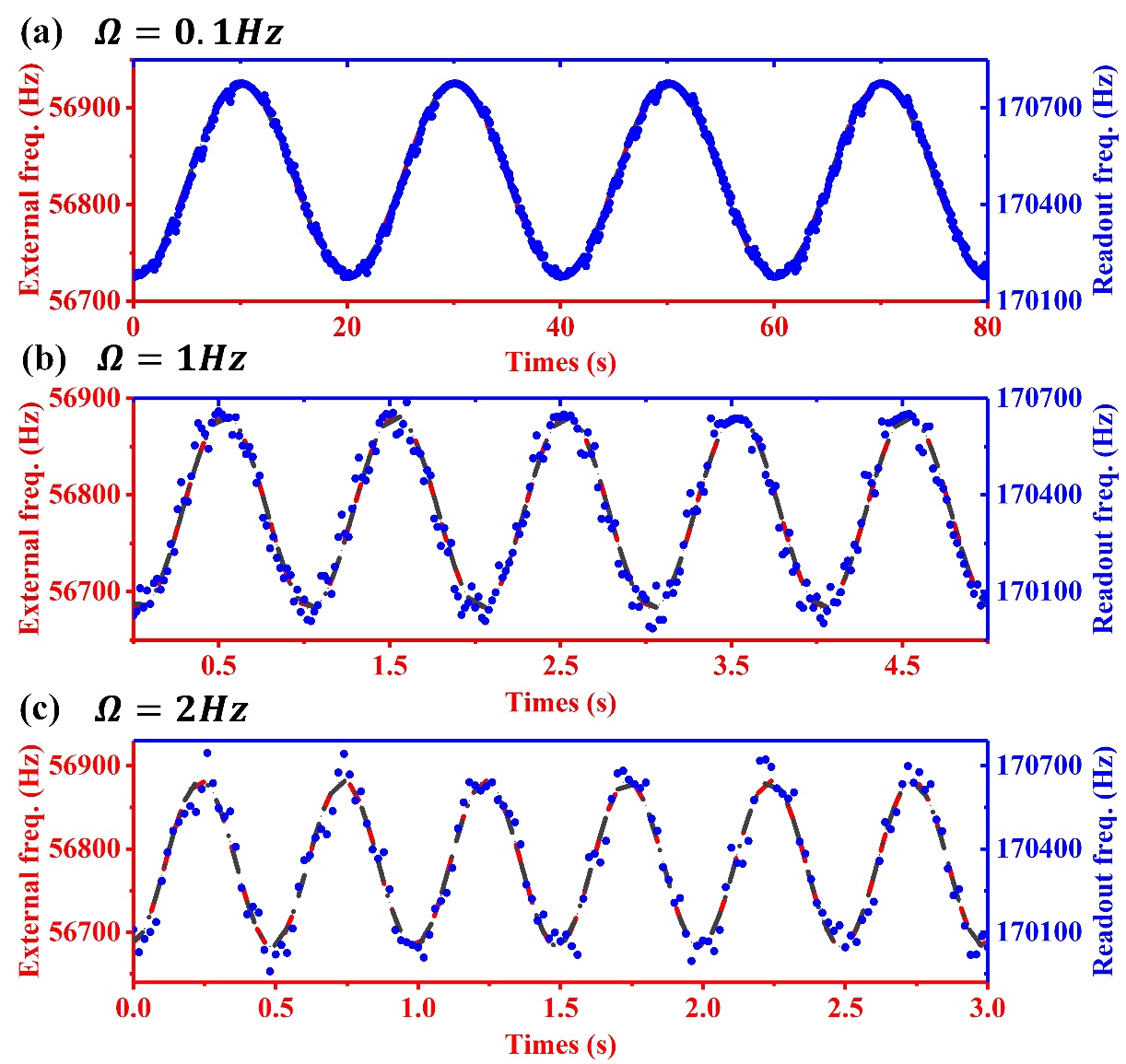


Figure S10. The experimental results of frequency tracking system for various dynamic frequencies. (a), (b) and (c) represent the output response of sensing oscillator and readout oscillator under dynamic modulation acceleration as 0.1Hz, 1Hz and 2Hz, respectively. The shorter tracking time will help to track higher frequency of modulation acceleration for automatic tracking system.
